# Differential regulation and correlation between galectin-9 and anti-CCP antibody (ACPA) in rheumatoid arthritis patients

**DOI:** 10.1186/s13075-020-02158-3

**Published:** 2020-04-15

**Authors:** Yuya Fujita, Tomoyuki Asano, Naoki Matsuoka, Jumpei Temmoku, Shuzo Sato, Haruki Matsumoto, Makiko Yashiro Furuya, Eiji Suzuki, Hiroshi Watanabe, Atsushi Kawakami, Kiyoshi Migita

**Affiliations:** 1 Department of Rheumatology, School of Medicine, Fukushima Medical University, 1 Hikarigaoka, Fukushima, Fukushima 960-1295 Japan; 2 Department of Immunology and Rheumatology, Unit of Advanced Preventive Medical Sciences, Nagasaki University Graduate School of Biomedical Sciences,, Nagasaki University, Sakamoto1-7-1, Nagasaki, 852-8501 Japan

**Keywords:** Anti-citrullinated peptide antibody, Galectin-9, Rheumatoid arthritis, T cell immunoglobulin domain and mucin-3

## Abstract

**Background:**

Galectin-9 (Gal-9) is involved in the regulatory process of immune responses or inflammation. The aim of the present study is to characterize circulating Gal-9 in patients with rheumatoid arthritis (RA) and its relationship with RA disease activity and phenotype.

**Methods:**

A total of 116 RA patients and 31 age-matched healthy controls were included in this study. Disease activity of RA patients was determined by Disease Activity Score of 28 joint scoring system (DAS28-ESR). Levels of Gal-9 in serum were determined by enzyme-linked immunosorbent assay (ELISA).

**Results:**

Serum levels of Gal-9 were significantly higher in patients with RA compared to those in controls (median 7577 pg/ml [interquartile range (IQR) 5570–10,201] versus 4738 pg/ml [IQR 4267–5630], *p* = 0.001). There were significant differences in serum Gal-9 between RA patients with and without RA-ILD (9606 pg/ml [IQR 8522–12,167] versus 7078 pg/ml [IQR 5225–9447], *p* < 0.001) or those with and without advanced joint damage (stage II–IV, 9606 pg/ml [IQR 8522–12,167] versus 7078 pg/ml [IQR 5225–9447], *p* < 0.001). Although serum levels of Gal-9 correlated with the titers of ACPA (*r* = 0.275, *p* = 0.002), levels of ACPA titers conferred the different relationship, between serum Gal-9 and inflammatory mediators or RA disease activity. Although Gal-9 was correlated with ACPA titers (*r* = 0.508, *p* = 0.002), there was no correlation between Gal-9 levels and erythrocyte sedimentation rate (ESR), matrix metalloproteinase-3 (MMP-3), or DAS28-ESR in RA patients with high titers of ACPA (> 200 U/ml). Conversely, Gal-9 was correlated with MMP-3 (*r* = 0.300, *p* = 0.007) or DAS28-ESR (*r* = 0.331, *p* = 0.004) but not with ACPA titer in RA patients with low titers of ACPA titers (< 200 U/ml).

**Conclusions:**

Serum levels of Gal-9 were increased in RA patients and associated with RA disease activity in RA patients without high titers of ACPA. The levels of ACPA titers may influence the values of circulating Gal-9 in RA patients with various clinical phenotypes. These data suggest that Gal-9 possessed the properties of pro-inflammatory or arthropathic biomarker under the status of ACPA titers.

## Introduction

Rheumatoid arthritis (RA) is an autoimmune disease characterized by synovial inflammation, bone destruction, and extra-articular symptoms [1]. Multiple genes, proteins, and cells have been identified to contribute to the pathogenesis of RA [2]. A common feature of RA is the hyper-activated state of the stromal synovial cells and the immune cells [3]. Dysregulated innate and adaptive pathways are involved in the RA pathogenesis [4]. Galectins are the family members of lectins that expressed on the cell surface or extracellular matrix and bind to β-galactoside carbohydrates on the cell surface [5]. Through binding to their receptors, galectins play an important role in the pathological processes including inflammation and autoimmunity [6]. Recent studies suggest that galectins play important roles in the pathogenesis of RA [7]. It was demonstrated that galectin-3 is increased in early RA and associated with anti-CCP seropositivity and MRI bone erosion scores in patients with RA [8]. These findings suggest that galectin family plays an important role in the disease development of RA, through their interactions with innate or adaptive immunity.

Gal-9 is expressed by immune cells, endothelial cells, and fibroblasts and plays an important role in regulating inflammation and immune reactions [9]. Gal-9 is a ligand of T cell immunoglobulin and mucin-containing-moleculte-3 (Tim-3) which is expressed on CD4^+^ T helper (Th) 1 and Th17 and providing inhibitory signals through its interaction with Tim-3 [10]. Therefore, Gal-9 negatively regulates pro-inflammatory T cell responses through the interaction with Tim-3 and Gal-9/Tim-3 pathway induces apoptosis of CD4^+^ Th1 or Th17 cells [11]. In mouse models, Gal-9 deficiency led to increased number of Th1 and Th17 cells and decreased number of Treg cells in the joint, rendering susceptibility to collagen-induced arthritis (CIA) [12]. Conversely, administration of Gal-9 ameliorated arthritis in CIA and immune complex-induced murine arthritis model suggesting that Gal-9 prevents the disease progression of RA [13]. Considering that RA is regarded as a Th1-polarized autoimmune disease, dysregulated Gal-9 levels may cause the imbalance in the innate/adaptive immunity, thereby inducing pathological rheumatoid inflammation. In inflammatory arthritis, Gal-9 was shown to mediate the angiogenesis and infiltrations of inflammatory cells [14]. These findings suggest that Gal-9 may contribute to the rheumatoid inflammatory processes. Therefore, we focused on Gal-9 and hypothesized that Gal-9 may play a role in the pathogenesis of RA. In this study, we examined the levels of serum Gal-9 in patients with RA and evaluated the results with respect to the clinical parameters.

## Methods

### Patients

This observational single-center study included 116 consecutive RA patients. Patients were enrolled between February 2012 and September 2019, with follow-up ending in September 2019. We retrospectively reviewed the records of these RA patients. All patients were treated in the Department of Rheumatology, Fukushima Medical University, from June 2009 to March 2019. All the patients met the 2010 ACR/EULAR classification criteria for the disease [15]. Probable RA or overlap syndromes were excluded.

The following clinico-demographic data were collected from the Medical Records Unit at Fukushima University Hospital: age, age at onset of RA, gender, Disease Activity Score-28 for Rheumatoid Arthritis with erythrocyte sedimentation rate (ESR) (DAS28-ESR) score [16], and extra-articular manifestations. High-resolution computed tomography (HRCT) imaging of the chest was evaluated by blinded radiologists to assess the evidence of RA-related interstitial lung disease (ILD) manifestations. This study was conducted in accordance with the principles of the Declaration of Helsinki. Ethical approval for this study (no. 2019097) was provided by the Ethics Committee of Fukushima Medical University.

### Measurement of clinical disease activity

All patients underwent clinical assessment at baseline, including 28-joint swollen and tender joint counts (28-SJC and 28-TJC, respectively), physician and patient global assessment with visual analogue scales (0–100 mm), and ESR (mm). The composite disease activity indices were subsequently calculated: DAS28-ESR [15]. Result of this score was reported in quantitative value divided into 4 categories: remission with score of < 2.6, mild activity if score of ≥ 2.6 to < 3.2, moderate activity if score of ≥ 3.2 to < 5.1, and high activity if score of ≥ 5.1. Serum MMP-3 levels were measured by latex immunoassay (Panaclear MMP-3 “Latex”; Sekisui Medical Company Limited, Tokyo, Japan). The patients’ anti-CCP antibodies were analyzed using commercially available second-generation chemiluminescent enzyme immunoassay kits (STACIA® MEBLux™ CCP test, Medical and Biological Laboratories, Aichi, Japan) according to the manufacturer’s instructions. The results were reported qualitatively where negative or positive for anti-CCP antibody was defined as < 20.0 U/ml or ≥ 20.0 U/ml, respectively. Radiographs were taken of both hands of each patient. Two rheumatologists, blinded to the patient’s identity and functional status, independently graded each hand radiographs and assigned as Steinbrocker radiographic stage [17].

### ELISA methods

Serum concentrations of galectin-9 were measured using enzyme-linked immunosorbent assay kit (R&D Systems, Minneapolis, MN, USA) according to the manufacturer’s instruction.

### Statistical analysis

Results were non-normally distributed and are presented throughout the manuscript with median and 25–75th centiles [median, IQR] and were compared by the Mann-Whitney *U* test. Correlations between continuous variables were analyzed by Spearman’s rank correlation test. All data entry and statistical analyses were performed using SPSS Statistics version 22.0 (IBM, Armonk, NY). In all the analyses, a 2-tailed *p* < 0.05 was considered statistically significant.

## Results

We recruited 116 patients with RA and 31 gender- and age-matched healthy subjects. The demographics and clinical characteristics of the RA patients are presented in Table 1. Among 116 patients, 83 patients (71.6%) were female. The median age of RA patients was 66 years. The majority of RA patients received DMARDs, mostly methotrexate or MTX in combination with other synthetic DMARDs. Despite the treatments, there was a median DAS28-ESR score of 2.8.

**Table 1:** Baseline characteristics of 116 Japanese patients with RA

Characteristics	Value
Age (years), median (IQR)	66 (56–73)
Female, n (%)	83 (71.6)
Smoker, n (%)	44 (37.9)
RA-ILD, n (%)	31 (26.7)
Duration of RA (year), median (IQR)	5 (2–10)
ESR (mm/h), median (IQR)	15.5 (7–27)
CRP (mg/dL), median (IQR)	0.29 (0.09–0.9)
MMP-3 (ng/mL), median (IQR)	106 (61.8–207.3)
RF (IU/mL), median (IQR)	44 (11.8–149.5)
Anti CCP-Ab (U/mL), median (IQR)	60.1 (4.0–373.1)
Corticosteroid, n (%)	53 (45.7)
Methotrexate, n (%)	59 (50.9)
Biologics, n (%)	38 (32.8)
DAS28-ESR, median (IQR)	2.8 (2.0–3.7)
Steinbrocker stage	I: 35, II:40, III 26, IV 13

*ILD* interstitial lung disease, *ESR* erythrocyte sedimentation rate, *CRP* C reactive protein, *MMP-3* matrix metalloproteinase-3, *RF* rheumatoid factor, *CDAI* Clinical Disease Activity index, *SDAI* simplified disease activity index, *DAS28* Disease Activity Score, *IQR* interquartile range

**Fig. 1:**
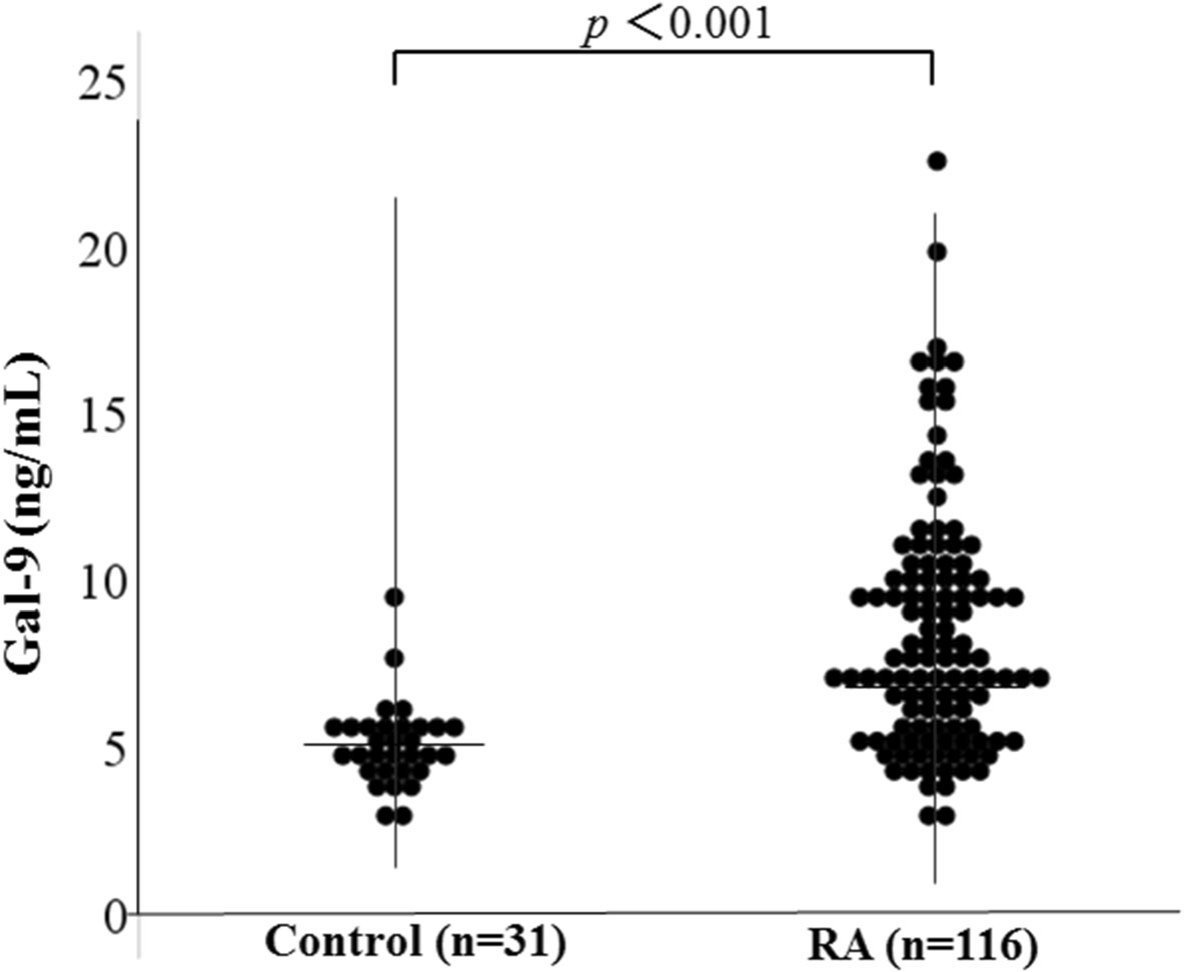
Serum levels of galectin-9 in RA patients and controls. Serum levels of galectin-9 in RA patients (*n* = 116) were significantly higher compared to those in healthy subjects (*n* = 31)

**Fig. 2:**
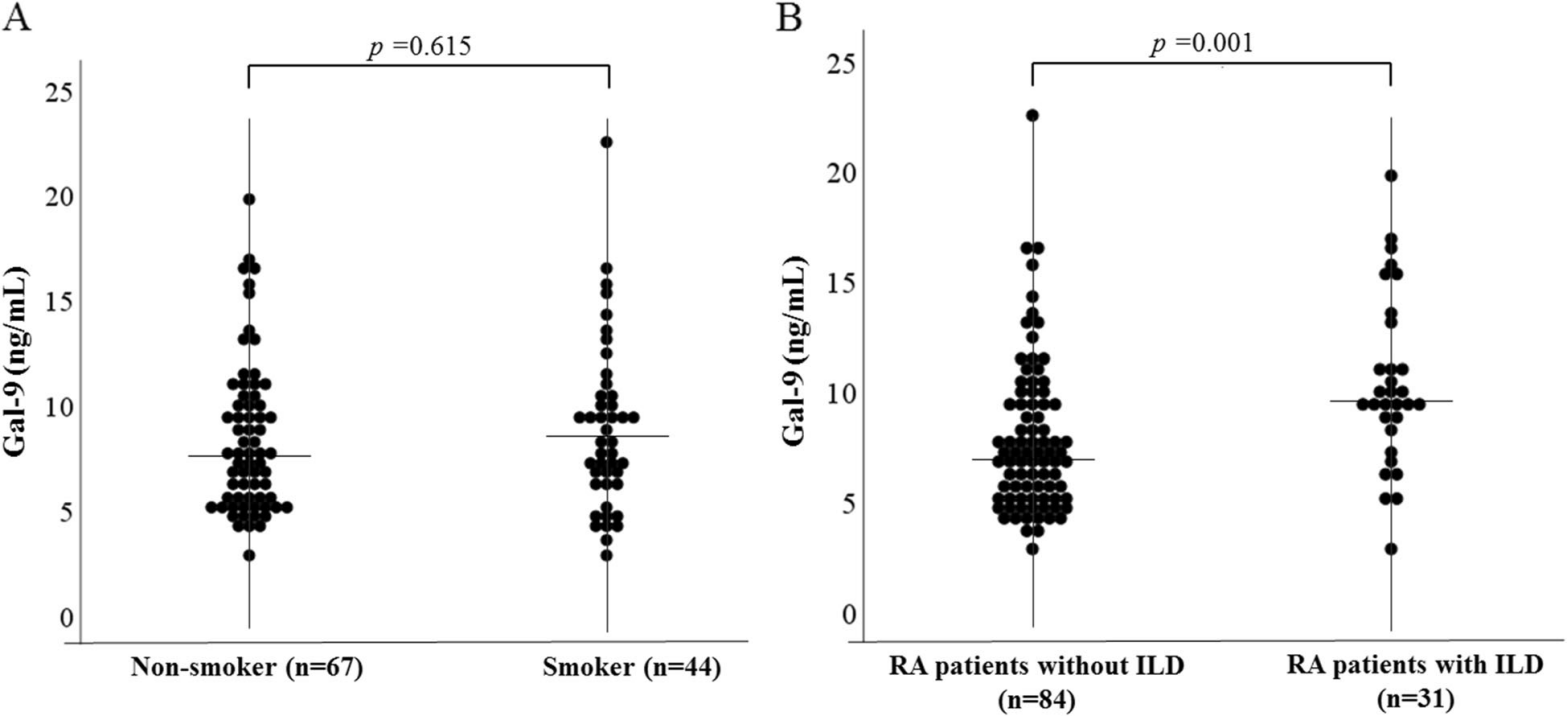
**a** Serum levels of galectin-9 in RA patients with or without RA-related interstitial lung disease (ILD). We compared serum levels of galectin-9 between RA patients with or without RA-related ILD. Serum levels of galectin-9 were significantly higher in patients with RA-related ILD compared to those without RA-related ILD. **b** Serum levels of galectin-9 in RA patients with or without smoking history. We compared serum levels of galectin-9 between RA patients with or without smoking history. There was no significant difference in serum levels of galectin-9 between RA patients with and without smoking history

We measured serum levels of Gal-9 in RA patients using a specific ELISA assay. As shown in Fig. 1, serum Gal-9 concentrations in patients with RA were significantly higher compared to those in healthy subjects (median 7577 pg/ml [interquartile range (IQR) 5570–10,201] versus 4738 pg/ml [IQR 4267–5630], *p* = 0.001). Serum levels of Gal-9 were compared in the subgrouped RA patients stratified by the disease durations. However, there was no significant difference in serum Gal-9 between RA patients with and without shorter disease durations (less than 5 years, 7009 pg/ml [IQR 5134–4527] versus 5 years or more, 7886 pg/ml [IQR 6154–10,692], *p* = 0.40). Although there was no significant difference in serum levels of Gal-9 between RA patients with and without smoking history (Fig. 2a, *p* = 0.615), higher levels of serum Gal-9 were observed predominantly in RA patients with RA-ILD (9606 pg/ml [IQR 8522–12,167] versus 7078 pg/ml [IQR 5225–9447], *p* < 0.001) (Fig. 2b). We also found a significant difference in ACPA titers between RA patients with and without RA-ILD (128.3 U/ml [IQR 24.7–896.0] versus 38.1 U/ml [IQR 2.5–215.2], *p* = 0.014).

**Fig. 3:**
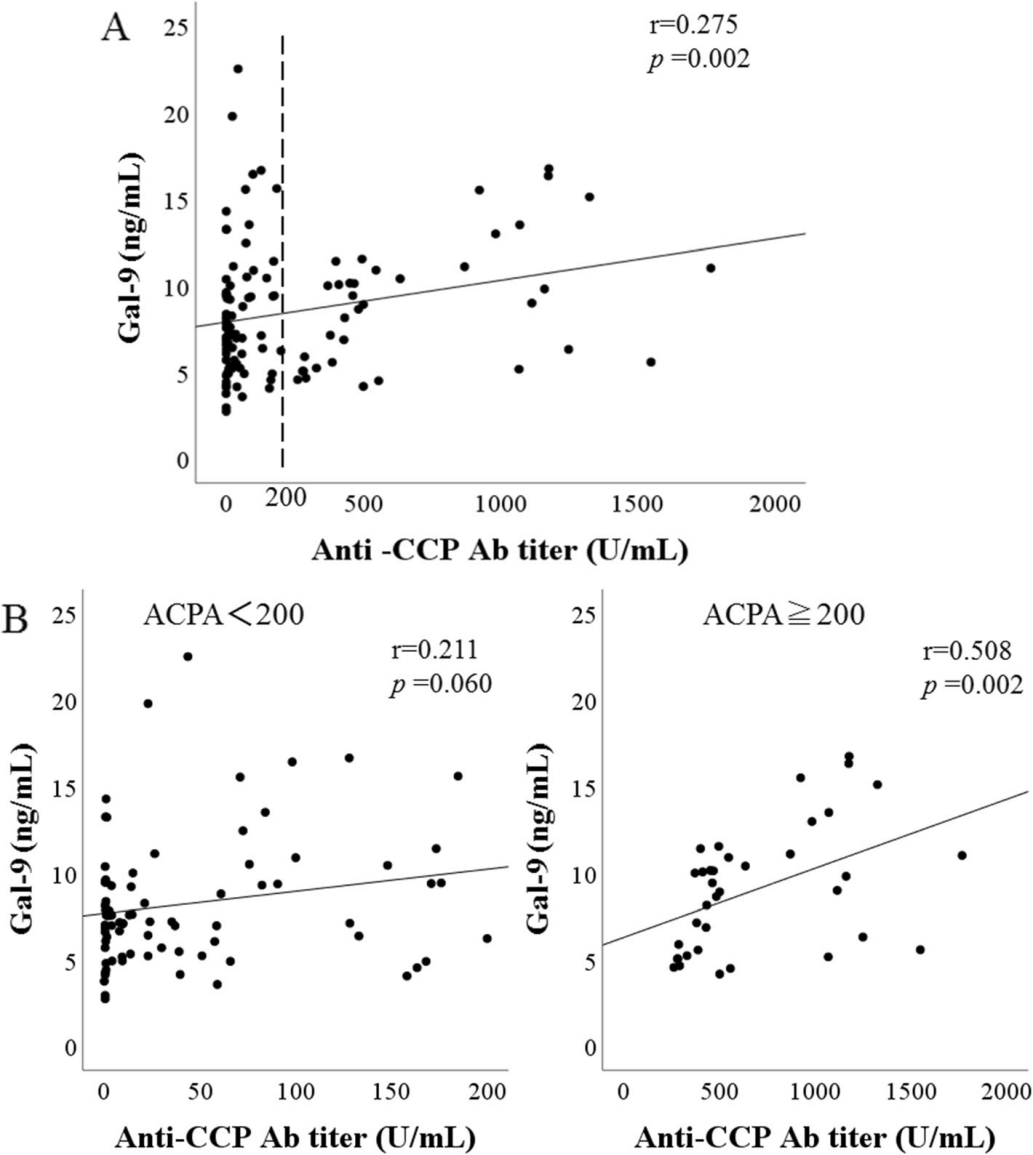
Relationship between anti-citrullinated peptide antibody (ACPA) titers and serum levels of galectin-9 in patients with rheumatoid arthritis (RA). **a** Levels of ACPA titers were measured, and correlation analysis with serum levels of galectin-9 was performed. **b** Correlation analysis of serum levels of galectin-9 and ACPA titers does not show a relationship in RA patients with low titers of ACPA (< 200 U/ml), whereas there was a significant positive correlation between serum levels of Gal-9 and ACPA titers in RA patients with high titers of ACPA (≧ 200 U/ml)

We investigated the relationship between serum Gal-9 and each parameter of RA patients (Fig. 3a). Serum Gal-9 were significantly correlated with ESR (*r* = 0.344, *p* < 0.001), MMP-3 (*r* = 0.234, *p* = 0.004), and ACPA titers (*r* = 0.275, *p* = 0.002). Also serum Gal-9 was significantly correlated with RA disease activity scores, DAS28-ESR (*r* = 0.269, *p* = 0.005).

Although we investigated the correlation between rheumatoid factor and serum Gal-9, there was no significant correlation between rheumatoid factor and Gal-9 (*r* = 0.16, *p* = 0.09, data not shown).

**Fig. 4:**
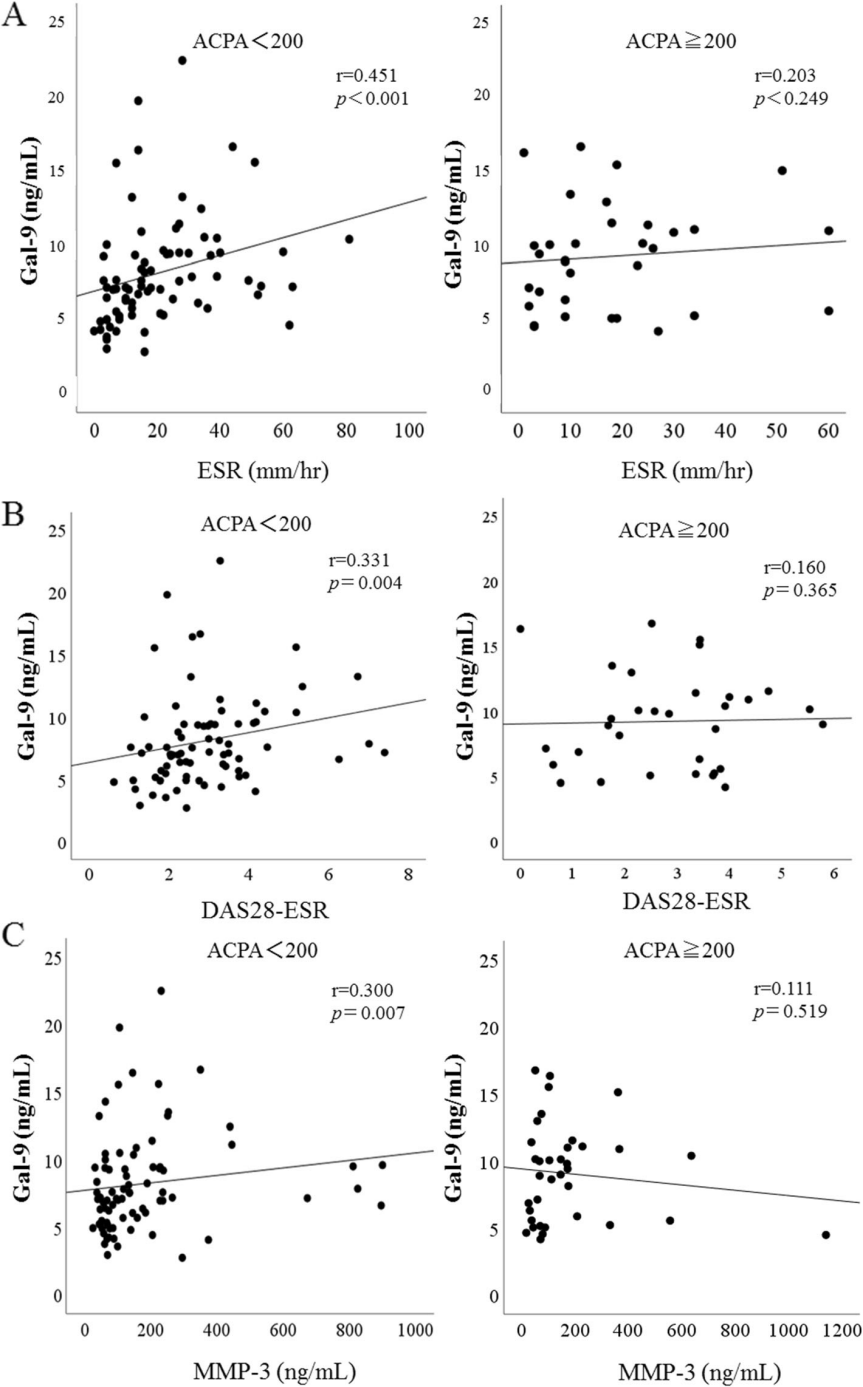
Correlation between serum levels of galectin-9 and clinical parameters (**a** ESR, **b** DAS28-ESR, **c** MMP-3) in the subgrouped RA patients according to the titers of ACPA. **a** Correlation analysis of serum levels of galectin-9 and ESR does not show a relationship in group 1 RA patients with high titers of ACPA (≧ 200 U/ml), whereas there was a significant positive correlation between serum levels of galectin-9 and ESR in group 2 RA patients with low titers of ACPA (< 200 U/ml). **b** Correlation analysis of serum levels of galectin-9 and DAS28-ESR does not show a relationship in group 1 RA patients with high titers of ACPA (≧ 200 U/ml), whereas there was a significant positive correlation between serum levels of galectin-9 and DAS28-ESR in group 2 RA patients with low titers of ACPA (< 200 U/ml). **c** Correlation analysis of serum levels of galectin-9 and MMP-3 does not show a relationship in group 1 RA patients with high titers of ACPA (≧ 200 U/ml), whereas there was a significant positive correlation between serum levels of galectin-9 and MMP-3 in group 2 RA patients with low titers of ACPA (< 200 U/ml)

To further evaluate the ability of serum Gal-9 to differentiate RA phenotype, we analyzed the distribution pattern of serum Gal-9 values in combination with ACPA titer (Fig. 3a). The cutoff values of ACPA titers (200 U/ml) were determined according to the ability to differentiate the inverse correlation between Gal-9 and ACPA titer. When RA patients were grouped according to the presence of high ACPA titers (≧ 200 U/ml), some correlations between circulating Gal-9 and clinical features were identified. In the two-dimensional heatmap consisting serum Gal-9 and ACPA titer, we identified two groups (Fig. 3a). Group 1 RA patients exhibited high ACPA titers (ACPA ≧ 200 U/ml), and group 2 RA patients exhibited moderate to low ACPA titers (ACPA < 200 U/ml). There was a significant modest correlation between Gal-9 and ACPA titers in group 1 RA patients (*r* = 0.508, *p* = 0.002). Conversely, there was no correlation between Gal-9 and ACPA titer in group 2 RA patients (*r* = 0.211, *p* = 0.060) suggesting that Gal-9 was not modulated by the status of ACPA titers (Fig. 3b). Next, we evaluated the correlations between Gal-9 and clinical parameters in subdivided group 1 or group 2 RA patients. Significant correlations between circulating Gal-9 and inflammatory markers, ESR (*r* = 0.451, *p* < 0.001) or DAS28-ESR (*r* = 0.331, *p* = 0.004), were identified in RA patients with low titers of ACPA (< 200 U/ml). However, there was no correlation between circulating Gal-9 and these parameters (*r* = 0.203, *p* = 0.249 and *r* = 0.160, *p* = 0.365) in RA patients with high titers of ACPA (≧ 200 U/ml) (Fig. 4a, b). Similarly, in RA patients with high titers of ACPA (≧ 200 U/ml), there was no correlation between serum Gal-9 and MMP-3 (*r* = 0.111, *p* = 0.519), whereas serum Gal-9 levels were significantly correlated with serum MMP-3 (*r* = 0.300, *p* = 0.007) in RA patients with low titers of ACPA (< 200 U/ml) (Fig. 4c). These findings suggest that serum Gal-9 were upregulated in link to autoimmune response in RA patients with high titers of ACPA. In contrast, serum Gal-9 was upregulated in response to inflammatory mediators in RA patients with low titers of ACPA.

**Fig. 5:**
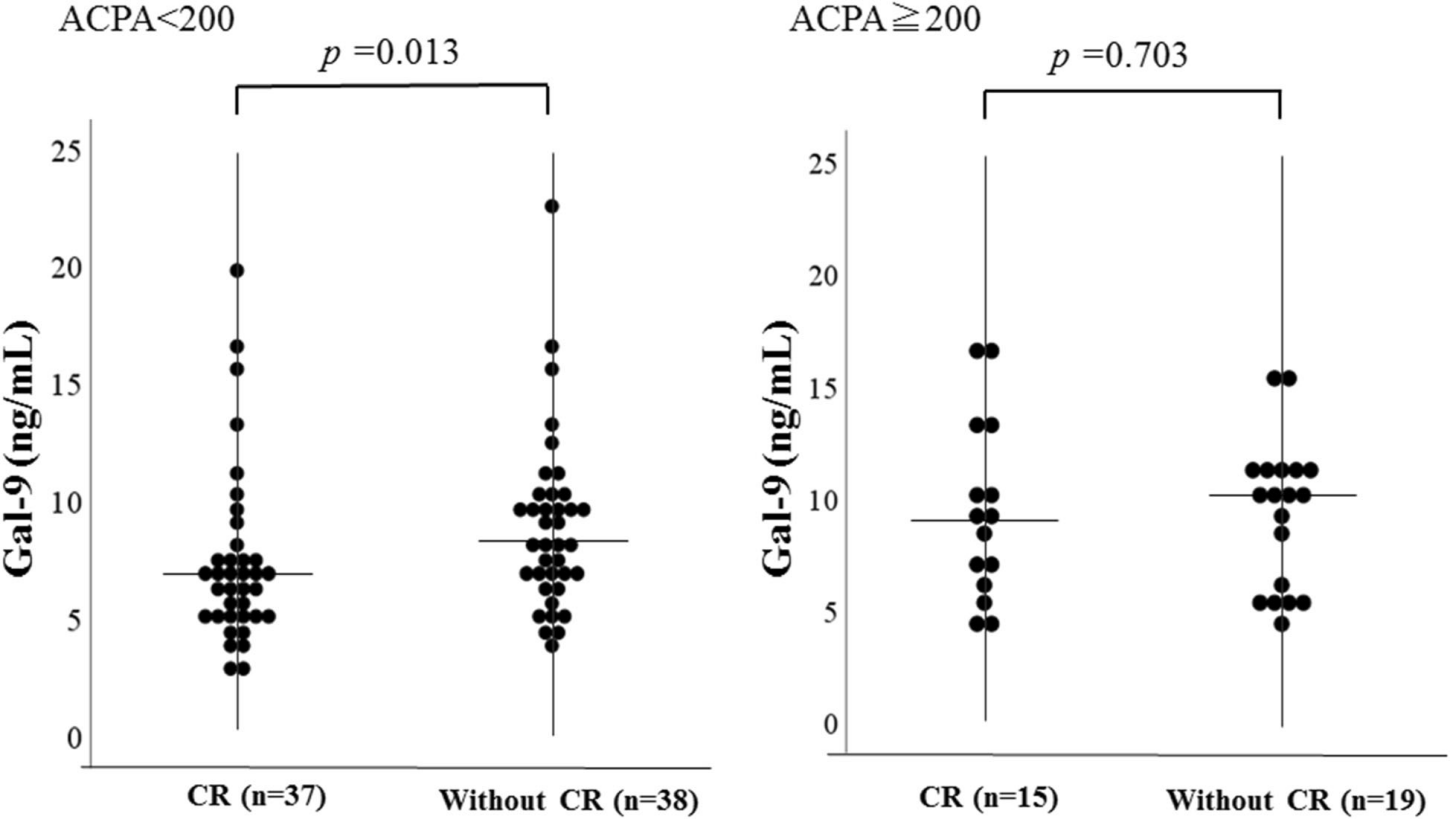
Serum levels of galectin-9 in RA patients with or without DAS28-ESR clinical remission (CR). We compared serum levels of galectin-9 between RA patients with or without clinical remission (CR). Serum levels of galectin-9 were significantly lower in patients with CR compared to those without CR in RA patients with low titers of ACPA (< 200 U/ml), whereas there was no significant difference in serum levels of galectin-9 between patients with and without CR in RA patients with high titers of ACPA (≧ 200 U/ml)

From a clinical point of view, the circulating Gal-9 were compared according to the presence or absence of clinical remission in these subgrouped RA patients (Fig. 5). In RA patients low titers of ACPA (< 200 U/ml), circulating Gal-9 were significantly higher in patients without clinical remission compared to those with clinical remission (8252 pg/ml [IQR 5870–10,996] versus 7103 pg/ml [IQR 5328–8357], *p* = 0.013). There was no significant difference in circulating Gal-9 between patients with and without clinical remission (10,647 pg/ml [IQR 6960–13,367] versus 8635 pg/ml [IQR 6372–10,092], *p* = 0.703) in RA patients with high titers of ACPA (≧ 200 U/ml).

**Fig. 6:**
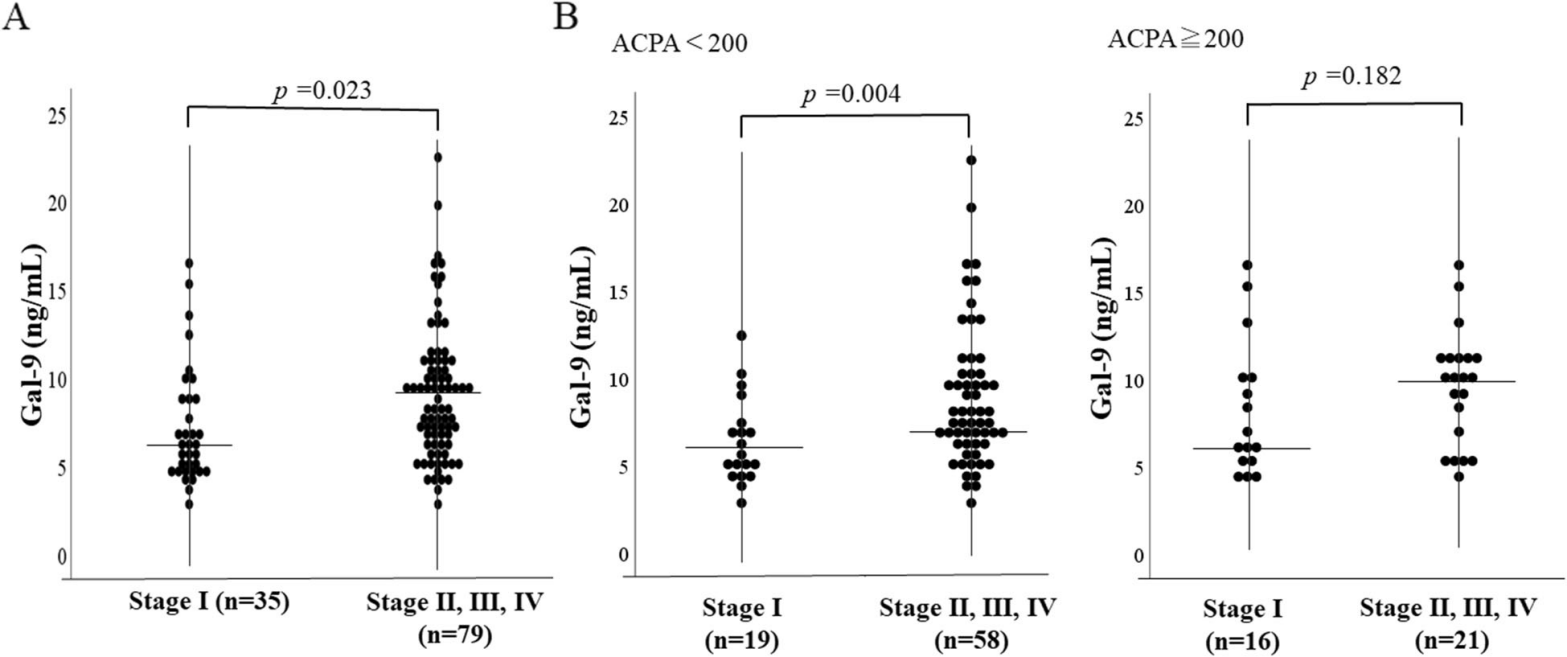
Serum levels of galectin-9 in RA patients with or without advanced joint damage. We compared serum levels of galectin-9 between RA patients with or without advanced joint damage (stage I versus stage II–IV). **a** Serum levels of galectin-9 were significantly higher in RA patients with advanced joint damage (stage II–IV) compared to those without advanced joint damage (stage I). **b** We compared serum levels of galectin-9 between RA patients with or without advanced joint damage (stage II–IV versus stage 1) according to the ACPA titers. Serum levels of galectin-9 were significantly higher in patients with advanced joint damage (stage II–IV) compared to those without advanced joint damage (stage I) in RA patients with low titers of ACPA (< 200 U/ml), whereas there was no significant difference in serum levels of galectin-9 between patients with and without advanced joint damage in RA patients with high titers of ACPA (≧ 200 U/ml)

Finally, we subdivided RA patients according to the progressing of joint damage (stage) and we evaluated the relationship between serum Gal-9 and progressive joint damage. As shown in Fig. 6a, RA patients with advanced articular lesions (stage II–IV) had significantly higher levels of circulating Gal-9 compared to those without advanced articular lesions (stage I) (8790 pg/ml [IQR 5631–10,953] versus 7103 pg/ml [IQR 5882–8810], *p* = 0.023). There was a significant difference in circulating Gal-9 between those with and without advanced articular lesions (stage II–IV) in RA patients with low titers of ACPA (7367 pg/ml [IQR 5931–10,109] versus 7009 pg/ml [IQR 4602–9450], *p* = 0.004). In contrast, there was no significant difference in circulating Gal-9 between those with and without advanced articular lesions (stage II–IV) in RA patients with high titers of ACPA (10,146 pg/ml [IQR 8771–12,257] versus 5882 pg/ml [IQR 5053–8138], *p* = 0.182) (Fig. 6b).

## Discussion

In this study, we evaluated circulating levels of Gal-9 in established RA patients with various disease activities. We demonstrated that Gal-9 is significantly elevated in RA patients and correlated with the titers of ACPA as well as rheumatoid inflammatory markers such as ESR or MMP-3. Conversely, we could not find positive correlations between Gal-9 concentrations and MMP-3 or DAS-28-ESR in subgrouped RA patients with high titers of ACPA (> 200 U/ml). These results suggest that galectin-9 could be a biomarker estimating RA disease activity under the particular ACPA status.

There is evidence that the pathogenesis of RA differs between ACPA-positive and ACPA-negative RA patients [18]. This antibody could be used as a prognostic factor, since high titers for ACPA are associated with worse radiographic progression [19]. It was also postulated that an association between seropositivity of ACPA and response to abatacept (ABT) could be caused by blocking the interaction between T cells and APC via costimulatory molecules in which the effects of ABT on T cell costimulation are more marked in ACPA-positive patients [20]. These data suggest that RA patients with high titer of ACPA represent a unique RA population.

We found a positive correlation between circulating Gal-9 and the inflammatory mediators in RA patients suggesting that the dysregulated circulating Gal-9 is involved in RA pathophysiology as a modulator for autoimmunity as well as rheumatoid inflammation. Furthermore, differential patterns of ACPA seropositivity and inflammatory mediators have implications in the increased circulating Gal-9 in RA patients. Gal-9 has been suggested to play a role in RA pathogenesis, but the underlying mechanisms have not been elucidated. Previous studies suggest that the measurement of disease activity alone is not sufficient to identify fast progressing RA and high titers of ACPA could be a risk factor for RA progression [21, 22]. The correlation between Gal-9 and ACPA observed in our study may suggest that high titers of ACPA may be linked to the rheumatoid inflammatory process through the Gal-9-mediated bone effects indirectly. Interestingly, we found increased serum levels of Gal-9 in RA patients with progressive joint damage. Galectin family is involved in the rheumatoid osteoclastogenesis and inflammatory bone destruction [23]. Expression of Tim-3 was demonstrated in osteoclasts, and Gal-9 markedly inhibited osteoclastogenesis via Tim-3/Gal-9 system [24]. Therefore, it is possible that the Tim-3/Gal-9 system may regulate the rheumatoid inflammatory bone destruction. Taken together, the elevated circulating levels of Gal-9 seen in RA patients may reflect the augmented status of osteoclastogenesis of RA joints. It was reported that unlike to exogenous Gal-9, endogenous Gal-9 is protective against apoptosis and enhances synovial fibroblast viability suggesting the pathogenic and pro-inflammatory role in RA [25]. Considering the complex biological role of Gal-9, further studies are needed to determine the mechanism for the increased Gal-9 expression and its function upon the disease activity and joint damage of RA patients.

More recently, Wiersma et al. have reported that serum Gal-9 levels were elevated and correlated with disease activity (DAS-28) or smoking status in RA patient [26]. Our data are almost in line with these data. Serum Gal-9 levels were elevated in RA patients with RA-related interstitial lung disease (RA-ILD); however, we could not find any associations between circulating Gal-9 and smoking status in our RA patients. Differences of ethnic and demographic data of the subjected RA patients may contribute to the differential result between our data and the reported data [26]. Further, large-scale investigations consisting of ethnic groups are needed to decide the mechanisms by which circulating Gal-9 is elevated in RA patients.

Variation of disease course and treatment response in RA patients originate from the heterogeneity of this syndrome [27]. ACPA-positive and ACPA-negative diseases have been shown to be associated with different genetic or environmental factors suggesting that different pathological mechanisms are implicated in these two separate disease subsets [4]. In our study, no significant correlation between Gal-9 levels and ACPA was observed in RA patients with low or moderate titers of ACPA (< 200 U/ml). However, serum Gal-9 levels were correlated with rheumatoid inflammatory markers, such as DAS-28 or MMP-3 in these patients. It was demonstrated that inflammatory bone destruction could be efficiently ameliorated by controlling the Tim-3/galectin-9 system [23]. Taken together, our data suggest that Gal-9 may be implicated in the joint damage in RA patients without high titers of ACPA (< 200 U/ml). It is also presumed that stratifying patients with RA on the basis of ACPA and Gal-9 status enables to identify more homogenous RA phenotype with respect to disease activity or joint structure damages.

There are several potential limitations of this study that should be considered. First, the patient population was relatively small and a larger study is essential to confirm our results. Second, all patients with RA and healthy individuals in this study were Japanese; additional studies in other ethnic groups are needed to verify these findings. Third, the mechanism by which Gal-9 contributes to the pathogenesis of RA was not clarified. Finally, it will be important to examine the longitudinal changes of serum galectin-9 levels in patients with RA and to assess their extra-articular involvements in the future studies. Nevertheless, our findings suggest that Gal-9 may be involved in the pathophysiology of RA reflecting disease activity or immune phenotypes of RA.

## Conclusions

Serum Gal-9 shows as an additional biomarker for evaluating disease activity in patients with RA. Prospective investigation of the combination of Gal-9 and ACPA may facilitate development of diagnostic tools to assess disease activity and disease phenotype in patients with RA.

## Data Availability

Not applicable

## Abbreviations

ACPA: Anti-citrullinated peptide antibodies
ESR: Erythrocyte sedimentation rate
Gal-9: Galectin-9
RA: Rheumatoid arthritis
MMP-3: Matrix metalloproteinase-3
